# New progress in imaging diagnosis and immunotherapy of breast cancer

**DOI:** 10.3389/fimmu.2025.1560257

**Published:** 2025-03-17

**Authors:** Jie He, Nan Liu, Li Zhao

**Affiliations:** ^1^ Department of Radiology, Sir Run Run Shaw Hospital, Zhejiang University School of Medicine, Hangzhou, Zhejiang, China; ^2^ Department of Translational Medicine and Clinical Research, Sir Run Run Shaw Hospital, Zhejiang University School of Medicine, Hangzhou, Zhejiang, China; ^3^ Department of Radiology, Shaoxing People’s Hospital, Shaoxing, Zhejiang, China

**Keywords:** breast cancer, PD-1, immune checkpoint, immunotherapy, radiotherapy, imaging diagnosis

## Abstract

Breast cancer (BC) is a predominant malignancy among women globally, with its etiology remaining largely elusive. Diagnosis primarily relies on invasive histopathological methods, which are often limited by sample representation and processing time. Consequently, non-invasive imaging techniques such as mammography, ultrasound, and Magnetic Resonance Imaging (MRI) are indispensable for BC screening, diagnosis, staging, and treatment monitoring. Recent advancements in imaging technologies and artificial intelligence-driven radiomics have enhanced precision medicine by enabling early detection, accurate molecular subtyping, and personalized therapeutic strategies. Despite reductions in mortality through traditional treatments, challenges like tumor heterogeneity and therapeutic resistance persist. Immunotherapies, particularly PD-1/PD-L1 inhibitors, have emerged as promising alternatives. This review explores recent developments in BC imaging diagnostics and immunotherapeutic approaches, aiming to inform clinical practices and optimize therapeutic outcomes.

## 1 Introduction

Breast cancer is one of the leading female malignancies globally and the second most prevalent cancer overall (1, 2). The etiology of BC remains unclear, with diagnoses primarily relying on morphological pathology, where histopathological examination is the gold standard (3). However, this invasive method faces limitations such as difficulty in obtaining representative samples and being time-consuming. Consequently, non-invasive imaging techniques like mammography, ultrasound, and MRI are essential for BC screening, diagnosis, staging, and monitoring treatment efficacy. Advances in imaging technologies and artificial intelligence have facilitated the emergence of radiomics, enhancing precision medicine through early detection, accurate molecular subtyping, staging, prognostic evaluations, and personalized treatment plans (4).

Traditional BC treatments include surgical removal, radiotherapy, chemotherapy, targeted therapies, and endocrine treatments, which have collectively reduced mortality rates. Nevertheless, challenges such as tumor heterogeneity, therapeutic resistance, metastasis, and disease recurrence persist, particularly in poor prognostic subtypes like HER-2 positive and triple-negative BC (TNBC). The five-year survival rate for advanced BC patients remains around 20% despite comprehensive treatments. There is an urgent need for innovative therapies, with immunotherapies gaining prominence. PD-1/PD-L1 inhibitors have shown effectiveness in BC immunotherapy, although challenges remain, especially for TNBC (5, 6). This review highlights recent advancements in imaging diagnostics and immunotherapy for BC to inform clinical interventions and therapeutic strategies.

## 2 BC imaging diagnosis

Imaging technologies are essential for the diagnosis, staging, and treatment monitoring of BC. Mammography is the primary screening tool, while ultrasound, including elastography and contrast-enhanced ultrasound (CEUS), aids in assessing tumor characteristics and lymph node involvement. MRI techniques like dynamic contrast-enhanced MRI (DCE-MRI), diffusion-weighted imaging (DWI), provide detailed insights into the tumor’s microenvironment, molecular subtypes, and response to therapy. Radiomics, combined with artificial intelligence and traditional imaging, enhances diagnostic accuracy and supports personalized treatment strategies.

### 2.1 Ultrasound imaging techniques

#### 2.1.1 Conventional ultrasound for BC detection

Conventional ultrasound is a cornerstone in BC screening, widely adopted due to its high sensitivity and specificity (7). The assessment of axillary lymph node metastasis is crucial for determining clinical outcomes and patient survival (8). Key ultrasound features, including tumor size, internal echotexture, margins, and Adler blood flow grading, are valuable for predicting axillary node involvement. Additionally, the longitudinal-to-transverse ratio serves as an important indicator for malignancy risk stratification, although its reliability in forecasting lymph node metastasis remains debated (9). Despite its critical role in diagnosing BC, guiding biopsies, localization, axillary evaluation, and follow-up, conventional ultrasound is associated with a high false-positive rate, resulting in numerous unnecessary biopsies. Studes found that recommendations for further assessment after the addition of ultrasonography to mammography screening approximately doubled, and biopsy recommendation rates increased 2- to 3-fold in patients with dense breast tissue (10, 11), potentially increasing the burden of misdiagnosis in these populations.

#### 2.1.2 Ultrasound elastography

Ultrasound elastography, including strain and shear wave elastography (SWE), enhances conventional ultrasound by assessing tissue stiffness to differentiate benign from malignant lesions, reducing unnecessary biopsies. The method applies external force to deform tissue, with the probe capturing displacement to generate grayscale or color images (12). Although breast elastography’s accuracy versus traditional B-mode ultrasound is debated, SWE lacks increased sensitivity for ductal/lobular carcinomas (13). Tumor size affects outcomes, with smaller lesions showing better sensitivity and specificity (14). Combining elastography with conventional ultrasound improves diagnostic performance (15, 16).

#### 2.1.3 CEUS

CEUS visualizes tumor contrast distribution, revealing microvascular architecture and blood supply, thereby differentiating benign from malignant breast tumors with 100% sensitivity and 87.5% specificity, strongly correlating with MRI findings (17). In BC subtype classification, Wen et al. (18) identified enhancement speed and intensity from 116 lesions as subtype indicators. CEUS effectively evaluates axillary lymph node malignancy. Niu et al. (19) found uniform enhancement benign and absent/weak enhancement malignant with high sensitivity. It also predicts sentinel lymph node metastasis (20), depicts lesion reduction and microvascular changes (21), and forecasts neoadjuvant chemotherapy response. Lee et al. (22) and Peng et al. (23) demonstrated CEUS’s superiority over MRI in predicting pathological response and residual tumor size. Thus, CEUS enhances diagnostic accuracy, aids subtype differentiation, evaluates lymph node status, and predicts chemotherapy outcomes, making it essential for post-treatment BC assessment.

#### 2.1.4 Automated breast ultrasound volume scanning

ABUS is a 3D ultrasound technique that automatically scans the breast from multiple angles, reducing operator dependence inherent in handheld methods. This enhances examination reproducibility and enables multi-planar reconstruction, with coronal views decreasing interpretation time (24). ABUS allows clinicians to review more images swiftly and demonstrates superior diagnostic performance for lesions smaller than 5 mm (25). However, it excludes axillary regions and lacks tools for assessing vascular distribution and tissue elasticity (26). Gatta et al. (27) showed that combining digital mammography with 3D prone-position ABUS significantly improves BC detection in women with dense breast tissue.

#### 2.1.5 S-detect technology

S-detect is a widely utilized AI-assisted system embedded in ultrasound machines, leveraging deep learning algorithms for computer-aided diagnosis. Aligned with the Breast Imaging Reporting and Data System (BI-RADS), it autonomously evaluates key tumor characteristics, including size, shape, depth, margins, and internal structure, and classifies findings as either “possibly benign” or “possibly malignant”. Applied in BC diagnostics, S-detect enhances ultrasound accuracy and clinical diagnostic capabilities (28, 29). When adjunctive for BI-RADS category 4 nodules, it significantly reduces false-positive biopsy rates, minimizing unnecessary invasive procedures (30).

#### 2.1.6 Ultrasound-guided percutaneous biopsy

Ultrasound-guided percutaneous biopsy employs real-time ultrasound to precisely locate lesions and guide needle insertion for tissue sampling, enabling accurate pathological examination. This minimally invasive, highly accurate, and low-trauma procedure is widely used in clinical practice (31). Pathological data confirm BC diagnoses and provide critical information on histological types, grading, and molecular tumor features, while also effectively detecting metastatic lymph nodes (32). In cases of ambiguous malignancies, surgical biopsy is often debated; preoperative malignancy assessment could reduce surgeries and patient burden. Girardi et al. (33) found that this technique enhances diagnostic accuracy for uncertain malignancies, with an upgrade rate of approximately 3%, thereby improving management of suspicious lesions.

### 2.2 MRI

#### 2.2.1 DCE-MRI

DCE-MRI employs high-resolution T1-weighted isotropic sequences and rapid gadolinium-based contrast agent administration via high-pressure injectors to enhance imaging. It exploits tumor-induced angiogenesis, leading to permeable blood vessels where contrast agents leak into the interstitial space, causing localized signal enhancement. By analyzing time-signal intensity curves, parameters such as K^trans^, k_ep_, and v_e_ differentiate enhancement kinetics in breast lesions. Quantitative MRI morphologies of invasive BC correlate significantly with immunohistochemical biomarkers and subtypes (34, 35). Differentiating benign from malignant lesions using K^trans^, k_ep_, and v_e_ achieves accuracy rates of 94.50%, 79.82%, and 87.16%, respectively, with sensitivities up to 99% and specificities as high as 97% (36).

#### 2.2.2 Magnetic resonance spectroscopy

MRS employs point-resolved spectroscopy or stimulated echo acquisition mode voxel sequences to acquire spectroscopic images for tissue chemical analysis (37). It demonstrates high diagnostic sensitivity and stable specificity, particularly effective for early-stage BC, small tumors, and non-mass enhancing lesions (38). Lipid metabolites differentiate benign from malignant conditions, enhancing MRI specificity in fat necrosis identification, reducing unnecessary biopsies. Invasive ductal carcinoma exhibits a higher water-to-fat ratio, indicating BC response to neoadjuvant chemotherapy (39, 40). MRS also evaluates tumor aggressiveness, with elevated total choline (tCho) in highly proliferative tumors and minimal choline peaks in low-activity lobular carcinoma (41). Despite its potential, MRS lacks widespread integration into multiparametric MRI protocols, requiring optimization through multicenter trials for reproducibility and accuracy.

#### 2.2.3 DWI

DWI measures the mobility of water molecules within tissues, indirectly reflecting pathological and physiological characteristics influenced by factors such as cellular density, membrane integrity, and microstructural constraints. It is clinically recognized as a highly sensitive method for BC detection (42). Research suggests that DWI may also aid in predicting pathological grading (43). Apparent Diffusion Coefficient (ADC) values are significantly reduced in both estrogen receptor (ER)-positive and ER-negative BC (44). Conversely, HER-2 positive BC exhibit higher ADC values compared to HER-2 negative cases. Interestingly, lower ADC values are observed in ER/PR-positive BC, which is atypical since ER/PR expression is generally associated with slower-growing, lower-grade tumors, indicating an area ripe for further investigation.

#### 2.2.4 Intravoxel incoherent motion diffusion-weighted imaging

IVIM imaging enhances DWI by using a bi-exponential model to separate microcirculatory perfusion from water diffusion. Parameters ADC, coefficient (D), pseudo-diffusion coefficient (D*), and perfusion fraction (F) differentiate benign from malignant breast lesions. Low b-values reflect both diffusion and perfusion, while high b-values mainly indicate diffusion, aiding tumor microcirculation and diffusion analysis (45). IVIM-MRI identifies tumor types, prognostic biomarkers, and therapy response. Lower ADC values link to aggressive invasive BC phenotypes (46, 47). In Luminal B tumors, D and ADC are lower than in Luminal A. ER expression correlates with ADC, D, and F, while D* relates to Ki-67. IVIM complements dynamic contrast-enhanced MRI for precise differentiation (48).

#### 2.2.5 Diffusion kurtosis imaging

DKI surpasses DWI and DTI by using non-Gaussian diffusion-weighted analysis, calculating diffusion coefficient (D) and kurtosis (K) to quantify tissue water diffusion deviations (49). It detects abnormal water diffusion in tissues, with invasive BC showing lower D values than benign lesions. Ductal carcinoma *in situ* also has lower D values than benign conditions. The 50th and 75th percentile D values in invasive BC are lower than in ductal carcinoma *in situ*, offering 95.7% specificity in distinguishing benign lesions from invasive cancer (49).

### 2.3 Mammography

2D digital mammography (DM) remains the leading modality for BC screening and diagnostic evaluation in recalled patients (50). However, DM’s efficacy is hindered by tissue overlap in 2D images, which diminishes sensitivity (70%, dropping to 30% in highly dense breasts) (51–53) and specificity (92%), causing 8% of healthy women to undergo unnecessary recalls (52, 54). Additionally, DM entails slight radiation exposure from x-rays (55) and significant patient discomfort due to required breast compression (56). Digital breast tomosynthesis (DBT) enhances mammography by acquiring multiple tomographic images per view, generating a “semi-3D” mammogram. This technique produces sequential thin slices, reducing tissue masking, improving cancer detection, and lowering false-positive rates (57).

### 2.4 Computed tomography imaging

Breast CT provides comprehensive three-dimensional imaging, thereby reducing the interference of overlapping anatomical tissues in breast evaluations. In this method, patients are positioned prone, allowing the breast to naturally extend away from the chest wall without compression. The x-ray source and flat-panel detector rotate horizontally around the breast, capturing numerous cone-beam projections that are subsequently reconstructed into a 3D CT image (58). While breast CT offers enhanced visualization of mass lesions compared to mammography (58, 59), it exhibits lower spatial resolution (60), is less effective in detecting microcalcifications (58), and involves a higher radiation dose.

### 2.5 Radiomics

Radiomics, introduced by Gillies et al. (61) extracts quantitative features from medical images using high-throughput computing, transforming images into multidimensional datasets for tumor evaluation, diagnosis, and prognosis prediction (62). The workflow includes data acquisition, tumor segmentation, feature extraction, selection, and model development (63, 64). In BC, radiomics applications are diverse: predicting axillary lymph node metastasis [Cui et (65)], combining mammography and MRI for sentinel lymph node prediction [Cheng et al. (66)], distinguishing BC subtypes and receptor status [Fan et al. (67), Leithner et al. (68)], assessing TILs (69), establishing immune scores [Han et al. (70)], and enhancing neoadjuvant chemotherapy (NAC) efficacy evaluation (71, 72). Radiomics also links MRI features with molecular subtypes, pathological complete response (pCR), and residual tumor burden [Choudhery et al. (73)], while predicting axillary metastasis and recurrence risk [Yu et al. (74) and Kim et al. (75)]. Ultrasound and PET radiomics further predict lymph node involvement, molecular subtypes, and recurrence (76–87). These advancements underscore radiomics’ potential as a non-invasive biomarker for precise BC clinical decision-making (**Table 1**).

**Table 1:** Overview of imaging technique for breast cancer diagnosis.

Category	Technique	Advantages	Disadvantages
Ultrasound Imaging	Conventional Ultrasound	High sensitivity and specificity; guides biopsies and evaluations.	High false-positive rate leading to unnecessary biopsies.
	Ultrasound Elastography	Reduces unnecessary biopsies; improves diagnostic accuracy when combined with conventional ultrasound.	Limited sensitivity for certain carcinoma types; effectiveness influenced by tumor size.
	Contrast-Enhanced Ultrasound (CEUS)	High sensitivity and specificity; correlates well with MRI; superior in certain predictive tasks.	Not explicitly mentioned.
	Automated Breast Ultrasound Volume Scanning (ABUS)	Reduces operator dependence; better for small lesions; enhances detection in dense tissue.	Does not assess axillary regions or tissue elasticity.
	S-detect Technology	Enhances diagnostic accuracy; reduces false-positive biopsies.	Not explicitly mentioned.
	Ultrasound-Guided Percutaneous Biopsy	Minimally invasive; highly accurate; provides essential diagnostic information.	May still require surgical biopsy in some cases.
Magnetic Resonance Imaging (MRI)	Dynamic Contrast-Enhanced MRI (DCE-MRI)	High accuracy in distinguishing lesions; correlates with molecular biomarkers.	Requires contrast agents; potential for false positives.
	Magnetic Resonance Spectroscopy (MRS)	High sensitivity; effective for early-stage and small tumors; reduces unnecessary biopsies.	Limited integration into standard protocols; needs further optimization.
	Diffusion-Weighted Imaging (DWI)	Highly sensitive for BC detection; provides insights into tumor biology.	Complex interpretation of ADC values; some paradoxical findings.
	Intravoxel Incoherent Motion DWI (IVIM)	Distinguishes tumor types; identifies prognostic markers; complements DCE-MRI.	Increased complexity in analysis and interpretation.
	Diffusion Kurtosis Imaging (DKI)	High specificity in differentiating benign from invasive cancer; detects complex diffusion patterns.	Requires advanced analysis techniques; not widely adopted.
Mammography	2D Digital Mammography (DM)	Widely available; high specificity (~92%).	Reduced sensitivity in dense breasts (~70%, down to 30%); radiation exposure; patient discomfort.
	Digital Breast Tomosynthesis (DBT)	Improves detection rates; lowers false positives; better visualization in dense breasts.	Not explicitly mentioned.
Computed Tomography (CT)	Breast CT	Better visualization of mass lesions compared to mammography.	Lower spatial resolution; less effective for microcalcifications; higher radiation dose.
Radiomics	Radiomics	Non-invasive biomarker; integrates multiple imaging modalities; supports precise clinical decisions.	Complex data analysis; requires specialized software and expertise.

## 3 Immunotherapy in BC

Immunotherapy aims to modulate the TME by targeting immune suppression and evasion, employing strategies such as antigen release, PD-1/L1 inhibition, immune activation, T cell infiltration, cancer recognition, and apoptosis induction (88–92), synergistically eliminating BC cells (93). However, the immunosuppressive TME remains a significant barrier to the efficacy of these therapies, especially in aggressive subtypes like TNBC.

### 3.1 Monotherapy with PD-1/PD-L1 inhibitors

PD-1, an immune checkpoint on activated cytotoxic T lymphocytes in the TME, maintains immune tolerance and limits tumor-facilitating responses (94–98). In BC, particularly in TNBC, tumors often overexpress PD-L1, which binds PD-1, inhibiting T cell proliferation and cytokine secretion, enabling immune evasion. Inflammation is pivotal in disease progression (99–102). The immunosuppressive TME, characterized by regulatory T cells (Tregs), myeloid-derived suppressor cells (MDSCs) (103), and tumor-associated macrophages (TAMs) secreting cytokines like TGF-β and IL-10, further suppresses T cell responses and promotes tumor progression (104–107). PD-1/PD-L1 inhibitors disrupt this interaction, reactivating T cells and suppressing MDSCs (108). However, monotherapy efficacy is limited: Avelumab shows 5.2% response in unselected TNBC and 22.2% in PD-L1^+^ cases (109), pembrolizumab achieves 18.5% in PD-L1^+^ TNBC (110), and other trials report ≤10% objective response rate (ORR) with no progression-free survival (PFS) or overall survival (OS) benefits (111–113). MDSC accumulation in TNBC further suppresses T cell activation, hindering PD-1/PD-L1 inhibitor efficacy (106). Combining these inhibitors with other immunomodulatory agents may enhance tumor antigen release, immune cell infiltration, and reduce immunosuppressive cell activity, potentially improving outcomes.

### 3.2 Combination immunotherapies

Combining ICIs, such as CTLA-4 inhibitors (e.g., ipilimumab) and PD-1/PD-L1 inhibitors, enhances antitumor immune responses by targeting distinct pathways. CTLA-4 inhibitors boost early T-cell activation, while PD-1/PD-L1 inhibitors prevent T-cell suppression in the TME (114, 115). This dual approach shows clinical efficacy across tumors, with metastatic BC achieving a 17% ORR (43% in TNBC, 0% in ER-positive) (116, 117). IDO inhibitors with nivolumab are also under investigation for advanced solid tumors, including BC (118).

### 3.3 Other immune-related therapies

Resistance to immunotherapy in BC often stems from the immune system’s inability to recognize tumors due to insufficient immunogenic neoantigens (119), which are crucial for personalized cancer vaccines (120). Cryoablation induces cell death, inflammation, and neoantigen exposure, enhancing immune detection (121). A pilot study in ER-positive patients demonstrated that ipilimumab combined with cryoablation significantly increased CD8^+^ T cells and Th1 cytokines, boosting antitumor immunity (122, 123). T cell receptor sequencing linked TILs to expanded T cell clones, serving as biomarkers (124). Post-translational modifications, like 5-azacytidine-induced hypomethylation of immune genes (125), and preclinical studies suggest PD-L1 upregulation (126). Histone deacetylase inhibitors also exhibit immunomodulatory effects (127), and combining epigenetic therapies with immunotherapies may improve antitumor responses and efficacy (128).

### 3.4 Combination with chemotherapy

Combining ICIs with chemotherapy results in higher TNBC response rates than ICI alone. Chemotherapy partially reverses the TNBC immunosuppressive microenvironment and upregulates PD-L1 on BC cells, creating synergy (129). A phase Ib trial of atezolizumab with albumin-bound paclitaxel in advanced TNBC showed a 39.4% ORR, likely due to paclitaxel-induced TLR activation and dendritic cell stimulation (130). The I-SPY2 trial indicated that adding anthracyclines and cyclophosphamide to paclitaxel and pembrolizumab increased efficacy from 22% to 60%, likely via anthracycline-induced immune stimulation and antigen presentation (131). Furthermore, KEYNOTE-355 found pembrolizumab with chemotherapy significantly improved PFS in PD-L1^+^ TNBC, especially CPS≥10 (132).

### 3.5 Combination with radiotherapy

By inducing antigen release, recruiting antigen-presenting cells, and stimulating T cell responses, radiotherapy enhances synergy with ICIs. In a BC mouse model, radiotherapy combined with PD-L1 blockade slowed tumor growth and activated CD8^+^ T cells (133). Clinical pilot studies showed partial responses and stable disease in HER2-positive and HER2-negative BC patients treated with ICIs and radiotherapy, with notable abscopal effects in metastatic TNBC patients (134). Despite small sample sizes, these findings underscore durable abscopal effects and the importance of patient selection, prompting further investigation in subsequent trials (135).

### 3.6 Combination with targeted therapy

Traditional targeted therapies in BC aim to improve patient survival by targeting oncogenes or tumor suppressor genes, though resistance often develops. Combining targeted therapies with immunotherapy can remodel the TME and enhance antitumor immune responses (136). PARP inhibitors targeting BRCA1/2 mutations elevate cytosolic DNA, activating interferon pathways and enhancing type I interferon and T cell infiltration (137). They also upregulate PD-L1, making them apt for immunotherapy combinations (138). The phase II TOPACIO trial reported a 47% response rate with niraparib and pembrolizumab (139), while ipatasertib with atezolizumab and paclitaxel achieved a 73% response (140). Ongoing studies assess MEK inhibitors with ICIs in TNBC, enhancing PD-1/L1 blockade responses (141).

### 3.7 Adoptive cell transfer therapies

**Figure 1:**
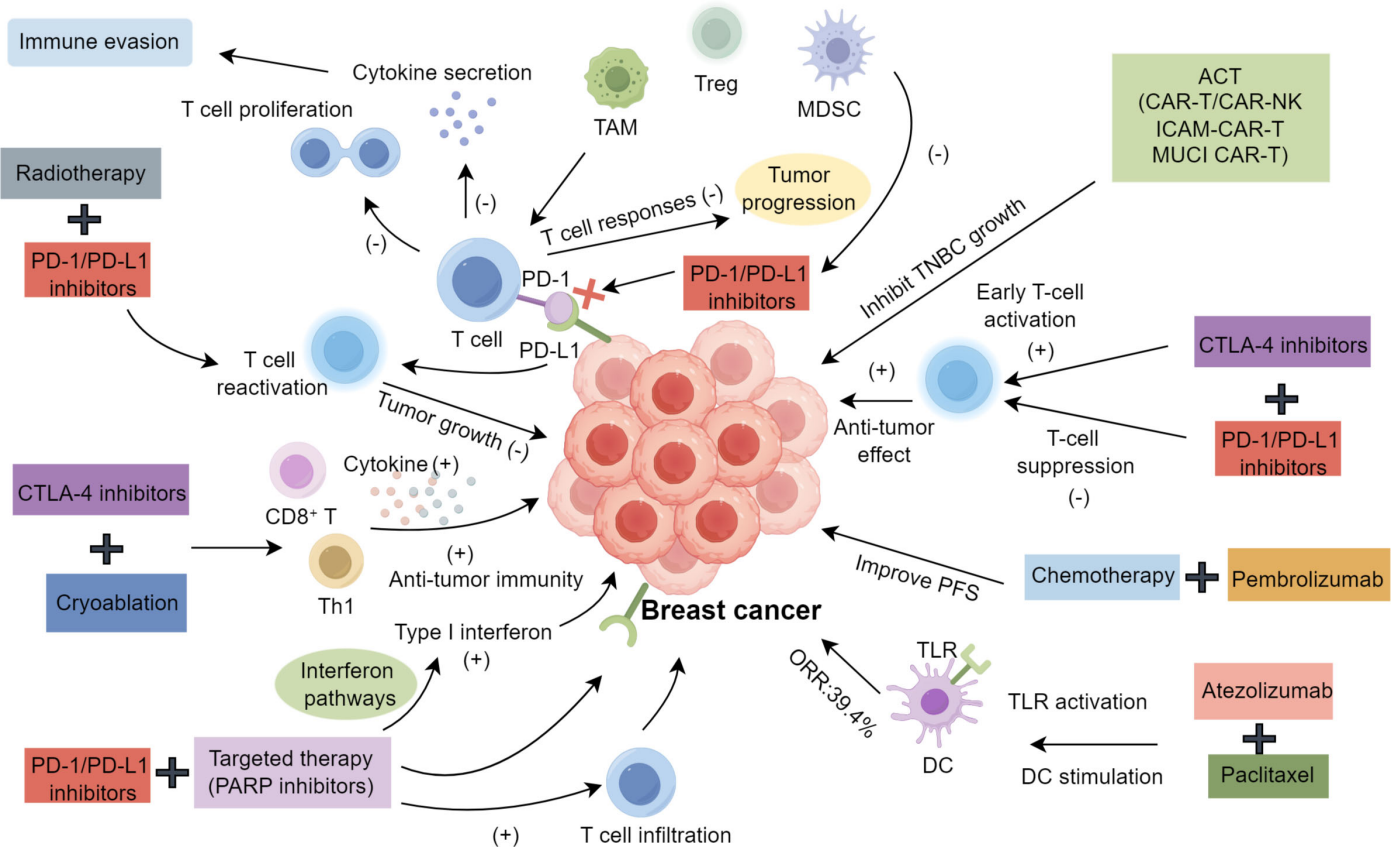
Immunotherapy in breast cancer.

Current ACT strategies for BC include TILs, CAR-T, CAR-NK, and TCR-T cells, each with distinct features. TILs act as prognostic indicators for BC outcomes. CAR-T and CAR-NK cells target solid tumors, with numerous clinical trials addressing various antigens. For example, EGFR-CAR-T cells significantly inhibit TNBC growth *in vitro* and *in vivo* (142), and ICAM-1-specific CAR-T cells effectively reduce tumor growth by targeting ICAM-1-expressing TNBC cells (143). MUCl-CAR-T therapy evaluated autologous MUCl-CAR-T cells in relapsed or refractory TNBC (144). CAR-NK therapies utilize NK receptors to induce apoptosis, showing therapeutic promise (145). While CAR-T trials in TNBC are expanding, TCR-T therapies are limited by MHC dependency (146) (**Figure 1**).

## 4 Conclusion

The integration of advanced imaging, radiomics, and artificial intelligence has enhanced BC diagnostics and molecular characterization. Immunotherapies, including PD-1/PD-L1 inhibitors and combination therapies, address tumor heterogeneity and resistance. Liquid biopsy, particularly circulating tumor DNA (ctDNA) detection, enables treatment monitoring and minimal residual disease detection (147). Nanotechnology improves drug delivery, enhancing efficacy and reducing side effects of chemotherapy and immunotherapy (148). Targeting the TME through immune checkpoint inhibition and immune suppression reversal offers transformative potential for BC treatment (149). Combined with genomic sequencing and data-driven models, these innovations promise a more precise and effective therapeutic framework for BC.
